# Effects of Pre-Meal Drinks with Protein and Amino Acids on Glycemic and Metabolic Responses at a Subsequent Composite Meal

**DOI:** 10.1371/journal.pone.0044731

**Published:** 2012-09-19

**Authors:** Ulrika J. Gunnerud, Cornelia Heinzle, Jens J. Holst, Elin M. Östman, Inger M. E. Björck

**Affiliations:** 1 Department of Applied Nutrition and Food Chemistry, Lund University, Lund, Sweden; 2 Department of Medical Physiology, The Panum Institute, University of Copenhagen, Copenhagen, Denmark; Clermont Université, France

## Abstract

**Background:**

Whey proteins have insulinogenic properties and the effect appears to originate from a specific postprandial plasma amino acid pattern. The insulinogenic effect can be mimicked by a specific mixture of the five amino acids iso, leu, lys, thr and val.

**Objective:**

The objective was to evaluate the efficacy of pre-meal boluses of whey or soy protein with or without added amino acids on glycaemia, insulinemia as well as on plasma responses of incretins and amino acids at a subsequent composite meal. Additionally, plasma ghrelin and subjective appetite responses were studied.

**Design:**

In randomized order, fourteen healthy volunteers were served a standardized composite ham sandwich meal with either water provided (250 ml) during the time course of the meal, or different pre-meal protein drinks (PMPD) (100 ml provided as a bolus) with additional water (150 ml) served to the meal. The PMPDs contained 9 g protein and were based on either whey or soy protein isolates, with or without addition of the five amino acids (iso, leu, lys, thr and val) or the five amino acids + arg.

**Results:**

All PMPD meals significantly reduced incremental area for plasma glucose response (iAUC) during the first 60 min. All whey based PMPD meals displayed lower glycemic indices compared to the reference meal. There were no significant differences for the insulinemic indices. The early insulin response (iAUC 0–15 min) correlated positively to plasma amino acids, GIP and GLP-1 as well as to the glycemic profile. Additionally, inverse correlations were found between insulin iAUC 0–15 min and the glucose peak.

**Conclusion:**

The data suggests that a pre-meal drink containing specific proteins/amino acids significantly reduces postprandial glycemia following a composite meal, in absence of elevated insulinemic excursions. An early phase insulinemic response induced by plasma amino acids and incretins appears to mediate the effect.

**Trial Registration:**

ClinicalTrials.gov NCT01586780<NCT01586780>

## Introduction

In type 2 diabetes (T2D), hyperglycemia is associated with an increased risk of cardiovascular diseases, and reduction of postprandial glycemia has recently been suggested to be as important as lowering fasting blood glucose levels to reach optimal metabolic control and reduce risk of complications [1]. Several epidemiological studies indicate that a high intake of milk and dairy products reduces the risk of developing T2D, obesity and cardiovascular disease [2]–[5], and a possible protective mechanism has been ascribed to the protein fraction [6]–[8]. In previous studies we have demonstrated that whey protein, co-ingested with carbohydrates, stimulates insulin secretion and reduces postprandial glycemia in both healthy subjects as well as in T2D patients [9], [10]. The insulinogenic effect appears to be mediated through a specific postprandial amino acid (AA) pattern appearing in plasma following whey ingestion, with the highest responses found for lysine (lys), threonine (thr) and the branched chained amino acids (BCAA) leucine (leu), isoleucine (iso) and valine (val) [10]. Furthermore, the release of the incretin glucose-dependent insulinotropic polypeptide (GIP) was proposed to be a contributing factor [10]. Interestingly, we have previously demonstrated that the insulinogenic effect can be mimicked by ingestion of a mixture of the mentioned 5 AA alone, however in the absence of a GIP response [11]. Additionally, oral administration of these 5 AA for 8 weeks has previously shown to improve insulin resistance on mice [12], suggesting possible beneficial effects of these particular 5AA in a longer time span.

It has been observed that intake of 18 g whey concentrate significantly increases insulin response and lowers glycemia compared with white wheat bread or glucose control [10], [11]. It could be hypothesized that exchanging part of the whey for insulinogenic AA (iso, leu, lys, thr and val) might be useful to optimize an insulinogenic effect. Furthermore, arginine (arg) has been suggested to increase insulin sensitivity [13], and also when co-ingested with leu, enhance the pancreatic insulin secretion [14]. Arg supplementation could therefore be expected to increase potency of the previously tested mixture of 5 AA. Soy protein is rich in arg and soy is often used as a substitute for milk and dairy products. Additionally, soy protein has been suggested to have beneficial effects on insulin resistance and obesity [15] as well on satiety [16]. The possible effect of soy protein on insulin response and glycemic regulation is therefore also of interest.

The aim of the present study was to evaluate the efficacy of pre-meal drinks of whey or soy protein isolates, respectively, with or without supplementation with AAs on insulinemia and glycemic regulation at a subsequent composite challenging meal. A mixture of five AA (iso, leu, lys, thr and val) was tested alone or in combination with arg. The potential timing effect was investigated based on the assumption that providing the insulinogenic proteins and AA as a pre-meal bolus would promote early insulin release, thus reducing the subsequent glycemic excursion. In addition, the effects on AA, incretins and ghrelin in plasma, as well as subjective satiety were investigated.

## Subjects and Methods

The protocol for this study and supporting CONSORT checklist and flow diagram are available as supporting information; see Protocol S1, Checklist S1 and Flow Diagram S1.

### Ethic Statement

The study was approved by the Ethics Committee in Lund, Sweden (reference number 556/2008). All test subjects gave their informed written consent prior to inclusion and were aware of the possibility of withdrawing from the study at any time they desired.

### Test Subjects and Study Design

14 healthy non-smoking volunteers (5 M, 9F) aged 20 to 28 y with normal body mass index (BMI; in kg/m^2^; 21.9±0.6; mean ± SEM) and not receiving any drug treatment participated in the study. All subjects had normal fasting plasma glucose concentrations (5.5±0.1 mmol/L; mean ± SEM) and no history of lactose malabsorption.

A randomized, single blind, within-subject trial was performed. The meals were provided as breakfasts on 7 different occasions in random order with approximately 1 week between each test. The subjects were instructed to eat a standardized meal, in the evening (between 21.00 and 22.00) prior to every test day, consisting of white wheat bread slices and an optional drink. Thereafter they were instructed to avoid eating and drinking anything but small amounts of water until the start of the test. Additionally they were asked to avoid alcohol, excessive physical activity and food rich in dietary fibers the day before each test. When the subjects arrived in the laboratory in the morning a peripheral catheter was inserted into an antecubital vein. After drawing a fasting blood (t = 0 min) sample the meals were served.

### Tests and Reference Meals

The nutritional compositions of all the meals are shown in **Table 1** **.** The meals were all based on one and the same standardized composite meal (ham sandwiches) with either 250 ml water provided during the time course of the meal, or different pre-meal protein drinks (PMPD) (100 ml) served as bolus doses with additional 150 ml water during the meal. The test subjects were instructed to drink the PMPD as a bolus immediately prior to eating the composite meal. Altogether, the PMPD and the sandwich meal were to be consumed within 12 min.

**Table 1:** Serving sizes and nutritional compositions of the reference and test meals^1^.

		Test drinks					
Meal components	Reference	W	W+5AA	W+6AA	S	S+5AA	S+6AA
	**g/serving**						
White wheat bread	106.4	106.4	106.4	106.4	106.4	106.4	106.4
Ham	27.7	27.7	27.7	27.7	27.7	27.7	27.7
Butter	10	10	10	10	10	10	10
Water served with the sandwich meal (mL)	250	150	150	150	150	150	150
Whey protein	―	9	4.5	4.5	―	―	―
Soy protein	―	―	―	―	9	4.5	4.5
Arginine	―	―	―	0.7	―	―	0.7
Isoleucine	―	―	0.7	0.7	―	0.7	0.7
Leucine	―	―	1.3	1.3	―	1.3	1.3
Lysine	―	―	0.9	0.9	―	0.9	0.9
Threonine	―	―	0.9	0.9	―	0.9	0.9
Valine	―	―	0.7	0.7	―	0.7	0.7
Water in the test drinks (mL)	―	100	100	100	100	100	100
∑ Amino acids	―	―	4.5	5.2	―	4.5	5.2
∑ Available carbohydrates	50.3	50.3	50.3	50.3	50.3	50.3	50.3
∑ Protein	13.3	22.3	22.3	23.0	22.3	22.3	23.0
∑ Fat	11.5	11.5	11.5	11.5	11.5	11.5	11.5
∑ Amount of water (mL)	250	250	250	250	250	250	250
∑ Energy (kcal)^2^	358	394	394	397	394	394	397

1 S (soy protein isolate); S+5AA (S, iso, leu, lys, thr and val); S+6AA (S+5AA and arg); W (whey protein isolate); W+5AA (W, iso, leu, lys, thr and val); W+6AA (W+5AA and arg).

2 Amount of energy per serving.

The PMPDs contained 9 g of protein, respectively and had the following composition; 1) whey protein isolate (W); 2) W, iso, leu, lys, thr and val (W+5AA); 3)W+5AA and arg (W+6AA); 4) soy protein isolate (S); 5) S, iso, leu, lys, thr and val (S+5AA); 6) S+5AA and arg (S+6AA).

All meals contained 50 g available carbohydrates in the form of white wheat bread (WWB). The protein content differed between the meals with the reference meal containing 13.3 g, and W, W+5AA, S and S+5AA containing 22.3 g of protein and the W+6AA and S+6AA meals had 23.0 g protein. The whey and soy protein isolates were kindly provided by Arla Foods Ingredients amba (Viby J. Denmark) and Semper AB (Stockholm, Sweden), respectively. The amino acid content of the whey and soy proteins is shown in **Table 2**. The whey protein contained maximum 0.2% lactose according to the manufacturer. All AA were single L-amino acids of food grade. Thr, iso and arg were manufactured by Ajinomoto (Kawasaki, Japan), whereas val, leu and lys were bought at Sigma-Aldrich AB (Stockholm, Sweden).

### Physiological Parameters and Subjective Rating of Appetite

Capillary blood samples were taken for plasma glucose analysis and venous blood was drawn for analysis of serum insulin, plasma ghrelin, free AA in plasma (p-AA), plasma glucagon-like peptide-1 (GLP-1) and GIP. However, GIP and GLP-1 were only analyzed for a subset of test meals; the reference, W, W+5 and the S meal, respectively. Moreover, the subjects were asked to repeatedly fill in their subjective feeling of fullness, hunger and desire to eat, respectively, using 100 mm Visual Analogue Scales (VAS). Glucose, insulin and subjective appetite ratings were measured at 0, 15, 30, 45, 60, 90, 120 and 180 min. Ghrelin was measured at 0, 30, 60, 90, 120 and 180 min, while p-AA were measured at 0, 15, 30, 45 and 60 min.

After sampling, capillary plasma glucose was analyzed immediately (Glucose 201+, Hemocue AB, Ängelholm, Sweden). Serum and plasma (EDTA) tubes were left on ice to rest for approximately 30 min before centrifugation for 10 min (1800 * g, 4°C). Thereafter, serum and plasma were immediately separated and the samples were frozen at −20°C until analysis. Insulin analysis was performed on an integrated immunoassay analyzer (CODA Open Microplate System; Bio-rad Laboratories, Hercules, CA, USA) using an enzyme immunoassay kit (Mercodia AB, Uppsala, Sweden). Ghrelin was analyzed using a radioimmunoassay kit (Linco research Inc., St. Charles, MO, USA). Free AA were purified by mixing 100 µl of 10% sulfosalicylic acid with 400 µl plasma to precipitate high-molecular-weight proteins according to the method of Pharmacia Biochrom Ltd (Cambridge, United Kingdom). The AA solutions were filtered before analyzing with an AA analyzer (Biochrom 30; Pharmacia Biochrom Ltd) by using ion-exchange chromatography. The AA were separated by using standard lithium citrate buffers of pH 2.80, 3.00, 3.15, 3.50 and 3.55. The postcolumn derivatization was performed with ninhydrin. The plasma concentration of aspartic acid and cysteine was close to the limit of detection and was therefore not evaluated.

Plasma GIP and GLP-1 concentrations were measured after extraction of plasma with 70% ethanol (by vol, final concentration). For the GIP radioimmunoassay [17], we used the C-terminal directed antiserum R 65, that cross-reacts fully with human GIP but not with so called GIP 8000, whose chemical nature and relation to GIP secretion is uncertain. Human GIP and ^125^I human GIP (70 MBq/nmol) were used for standards and tracers. The concentration of plasma GLP-1 was measured [18] against standards of synthetic GLP-1 7–36 amide by using antiserum code no. 89390, which is specific for the amidated carboxyl terminus of GLP-1 and, therefore, does not react with GLP-1 containing peptides from the pancreas. For both assays the sensitivity was <1 pmol/L, intraassay CV <6% at 20 pmol/L, and recovery of standard, added to plasma before extraction, ≈100% when corrected for losses inherent in the plasma extraction procedure.

**Table 2:** Content of amino acids in the whey and soy protein isolates.

Amino acids	Whey	Soy
	**g AA/100 g protein**	
Alanine	5.5	3.8
Arginine	2.1	8.1
Aspartic acid	11.9	11.3
Cystein	2.7	―
Glutamine	19.5	19.2
Glycine	1.8	3.7
Histidine	2.0	2.3
Isoleucine	7.6	―
Leucine	11.4	9.2
Lysine	10.4	6.3
Methionine	2.4	1.3
Phenylalanine	3.2	5.7
Proline	7.3	4.8
Serine	5.7	4.6
Threonine	8.1	3.6
Tryptophan	2.0	―
Tyrosine	3.1	4.0
Valine	6.6	5.0

### Calculations and Statistical Methods

The results are expressed as means ± SEM and values of p≤0.05 were considered statistically significant. Fasting values of glucose, insulin, p-AA, ghrelin, GLP-1 and GIP were analyzed with a general linear model (ANOVA) using subject as a random variable. The incremental areas under the curve (iAUC) for glucose, insulin GIP, GLP-1and p-AA as well as the total areas under the curve (AUC) for ghrelin and the subjective appetite ratings were calculated for each subject and test meal, using the trapezoid model (GraphPad Prism, release 5.03, GraphPad software Inc, San Diego). The glycemic and insulinemic indices (GI and II) were calculated from the 120 min postprandial iAUC for plasma glucose and serum insulin, respectively, by using the ham sandwich meal as a reference (GI and II = 100). Incremental peaks (iPeak) for glucose and insulin were calculated as the maximum postprandial increase from baseline. The glycemic profile (GP), defined as the duration (up to 180 min) of the glucose curve divided with the iPeak of glucose, was calculated [19]. In cases where the glucose remained above fasting level for the entire 180 min, the duration value was set to 180 min. The insulinogenic index (IGI), defined as the insulin iAUC 0–45 min divided with the glucose iAUC 0–45 min was calculated. A high insulinogenic index (IGI; nmol/mmol) represents a high potential of insulin to lower glucose within the first 45 min postprandially [20]. For ghrelin, relative changes from nadir to the concentration at 180 min after commencing the test meals as well as the concentration at 180 min and the AUC 0–180 min were calculated, using the trapezoid model.

The data were analyzed using a mixed model ANCOVA with subject as a random variable and the corresponding baseline (fasting values) as a covariate. Differences between groups were identified using Tukey’s multiple comparisons tests (MINITAB, release 14, Minitab Inc., State College, PA). In the cases of unevenly distributed residuals (tested with Anderson-Darling test), Box-Cox transformations were performed on the data prior to the ANOVA and/or ANCOVA, respectively.

Time x treatment interactions for glucose, insulin, subjective appetite ratings, ghrelin, GIP and GLP-1 as well as p-AA responses were analyzed using a mixed model (PROC MIXED in SAS release 8.01, SAS institute Inc., Cary, NC) with repeated measures and an autoregressive covariance structure. Subjects were modeled as a random variable and corresponding baseline values (fasting values) were modeled as covariate. When significant interactions between treatment and time were found, Tukey’s multiple comparisons test were performed for each time point by using the MINITAB software.

Correlation analysis was conducted to evaluate the relation among dependent measures with the use of Spearman’s partial correlation coefficients controlling for subjects and corresponding baseline values (two-tailed test), (SPSS software, version 18; SPSS Inc., Chicago, IL, USA).

Due to a standard preparation mistake during some of the AA analysis, a correction using a reduction of the concentrations by 22% was necessary. One subject did not consume the W+6AA and S+6AA meals and is therefore not included in the analysis on these two meals. One subject had to be excluded from the S meal due to reported inappropriate behavior the day prior to the test day. One more subject was excluded from the W+5AA and W+6AA analysis due to the intake of antibiotics within two weeks before the test days. Due to the same reason another subject was excluded from the data analysis on S+6AA. One subject was also excluded from the insulinogenic index (IGI) on W+5AA, caused by a glucose area equal to zero at AUC 0–45, leading to an incalculable IGI. Furthermore, two subjects were excluded from the ghrelin due to missing values, one from the reference meal and one from the S.

## Results

### Plasma Glucose Responses

**Figure 1:**
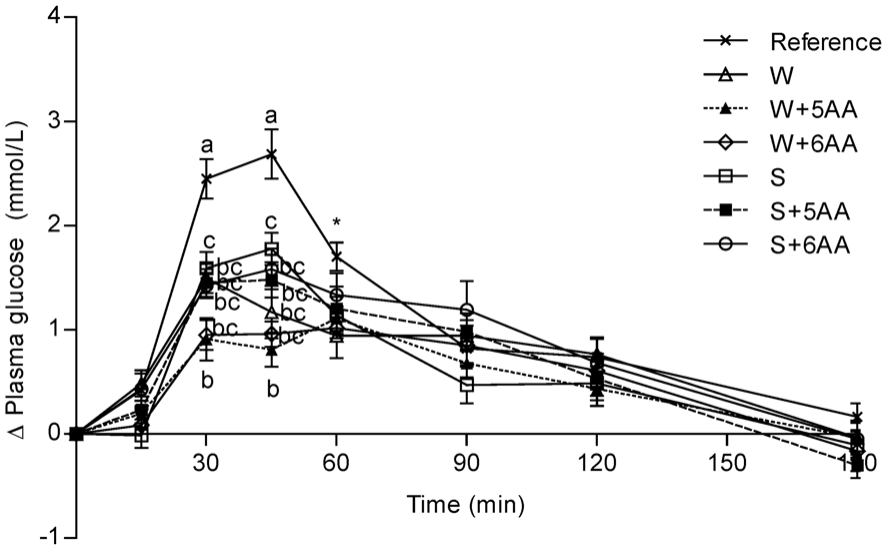
Postprandial plasma glucose responses. Mean (± SEM) incremental postprandial changes (Δ) in plasma glucose in response to equal amounts of carbohydrates from a reference meal and the PMPD meals; n = 14 (n = 13 for S and S+6AA; n = 12 for W+6AA). Values with different letters are significantly different, P≤0.05 (ANCOVA followed by Tukey’s multiple comparisons test). A significant treatment effect (P<0.001) and a significant time x treatment interaction (P<0.001) was found. *At 60 min W induced a significantly lower glucose response than did the reference. S (soy protein isolate); S+5AA (S, iso, leu, lys, thr and val); S+6AA (S+5AA and arg); W (whey protein isolate); W+5AA (W, iso, leu, lys, thr and val); W+6AA (W+5AA and arg).

The postprandial glucose responses are shown in **Figure 1** and **Tables 3** and **4**. The three whey based PMPD (WB-PMPD) meals but not the soy based PMPD (SB-PMPD) meals displayed lower GIs (%) compared to the reference meal. All PMPD meals resulted in a significantly lower postprandial glucose response (iAUC) during the first 45 min **(** **Table 4** **)**. A significant treatment (P**<**0.0001) and time x treatment interaction (P**<**0.0001) was found, and at 30 and 45 min, the glucose responses were lower following all PMPD meals compared to the reference meal. Furthermore, at 30 min the glucose response to S was higher than W+5AA, and at 45 min the glucose response of the S meal was higher than W+5AA as well as W+6AA. Additionally, the iPeak for glucose (Δ mmol/L) was lower following all PMPD meals in comparison to the reference. Moreover, the glycemic profile (GP; min.mmol^−1^.L^−1^.) was higher for the W, W+5AA, W+6AA and S+5AA meals, respectively, than for the reference meal (**Table 3**). The WB-PMPD meals induced lower GI’s (mean 61±9), higher GP’s (mean 114±15) and lower glucose iPeaks (mean 1.5±0.1) in comparison to the SB-PMPD meals (mean GI 74±12; mean GP 82±10; mean iPeak 1.9±0.2).

**Table 3:** Plasma glucose and serum insulin responses after the meals^1^.

Meals	GI^2^	iPeak glucose	GP	II^2^	iPeak insulin	IGI
	%	Δ mmol/L	min.mmol^−1^.L^−1^.	%	Δ mmol/L	(0–45 min iAUC)
Reference	100^a^	2.9±0.2^a^	55±5^b^	100^a^	0.35±0.04^a^	0.13±0.02^e^
W	73±11^b^	1.7±0.2^b^	89±7^a^	118±10^a^	0.38±0.04^a^	0.27±0.03^abd^
W+5AA	53±8^b^	1.4±0.1^b^	127±22^a^	123±14^a^	0.38±0.04^a^	0.60±0.18^d^
W+6AA	60±6^b^	1.4±0.1^b^	125±16^a^	121±10^a^	0.34±0.04^a^	0.47±0.13^bd^
S	67±9^ab^	1.9±0.1^b^	78±7^ab^	105±13^a^	0.31±0.03^a^	0.20±0.02^ce^
S+5AA	74±9^ab^	1.8±0.2^b^	88±12^a^	119±9^a^	0.38±0.05^a^	0.26±0.03^bc^
S+6AA	91±18^ab^	1.9±0.2^b^	81±11^ab^	123±9^a^	0.39±0.03^a^	0.25±0.04^ac^

1 All values are mean ± SEM. Values in the same column with different superscript letters are significantly different, P≤0.05 (ANCOVA followed by Tukey’s multiple comparisons test); n = 14 (n = 13 for S and S+6AA; n = 12 for W+6AA).

2 GI and II are expressed as iAUC 0–120 min.

GI, glycemic index; GP, glycemic profile; IGI, insulinogenic index; II, insulinemic index; S (soy prpone.0044731.g004.tifotein isolate); S+5AA (S, iso, leu, lys, thr and val); S+6AA (S+5AA and arg); W (whey protein isolate); W+5AA (W, iso, leu, lys, thr and val); W+6AA (W+5AA and arg).

**Table 4:** Early postprandial incremental areas under the curve (iAUC) for plasma glucose and serum insulin responses^1^.

	Glucose			Insulin
Meals	iAUC (0–30 min)	iAUC (0–45 min)	iAUC (0–60 min)	iAUC (0–30 min)
Reference	25.0±2.9^a^	63.5±4.7^a^	96.4±6.4^a^	3.3±0.6^ac^
W	18.8±2.6^ab^	38.7±4.8^b^	54.7±7.8^b^	4.8±0.7^abc^
W+5AA	11.4±2.5^b^	24.5±4.4^b^	39.0±5.7^b^	5.2±0.6^b^
W+6AA	9.9±2.1^b^	24.3±3.0^b^	39.1±3.6^b^	4.5±0.6^abc^
S	13.4±1.2^ab^	38.7±3.8^b^	60.6±6.1^ab^	3.2±0.4^a^
S+5AA	14.9±1.9^ab^	37.8±3.8^b^	57.0±6.1^b^	4.4±0.5^abc^
S+6AA	17.7±3.2^ab^	40.2±4.3^b^	62.1±6.3^ab^	4.3±0.6^abc^

1 All values are mean ± SEM; n = 14 (n = 13 for S and S+6AA; n = 12 for W+6AA). Values in the same column with different superscript letters are significantly different, P≤0.05 (ANCOVA followed by Tukey’s multiple comparisons test). S (soy protein isolate); S+5AA (S, iso, leu, lys, thr and val); S+6AA (S+5AA and arg); W (whey protein isolate); W+5AA (W, iso, leu, lys, thr and val); W+6AA (W+5AA and arg).

### Serum Insulin Responses

**Figure 2:**
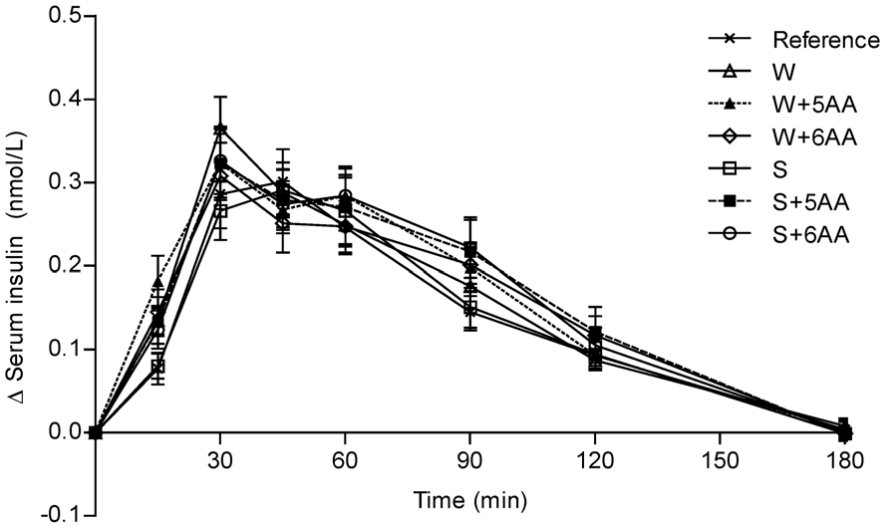
Postprandial serum insulin responses. Mean (± SEM) incremental postprandial changes (Δ) in serum insulin in response to equal amounts of carbohydrates from a reference meal and the PMPD meals; n = 14 (n = 13 for S and S+6AA; n = 12 for W+6AA). A significant treatment effect (P<0.001) but no time x treatment interaction was found. S (soy protein isolate); S+5AA (S, iso, leu, lys, thr and val); S+6AA (S+5AA and arg); W (whey protein isolate); W+5AA (W, iso, leu, lys, thr and val); W+6AA (W+5AA and arg).

The insulin responses are shown in **Figure 2** and **Tables 3** and **4**. In the early postprandial phase, expressed as iAUC 0–30 min, the W+5AA resulted in a higher insulin response compared to the reference and S, respectively (**Table 4**). No significant differences were found between the meals neither in insulinemic index (II in %) nor in iPeak for insulin (Δ mmol/L) (**Figure 2**; **Table 3**). All PMPDs except for S induced higher IGI’s than the reference meal. Moreover, the WB-PMPD meals displayed higher IGIs (mean 0.45±0.11) compared to the SB-PMPD meals (mean 0.24±0.03) (**Table 3** **)**.

### Postprandial Plasma Amino Acid Responses

Except for asp, no differences were found in fasting p-AA concentrations. The postprandial iAUC 0–60 min are displayed in **Table 5**. The plasma responses of arg, iso, leu, lys, and val were higher following all PMPD meals compared to the reference meal. Thr was higher after all PMPD except for the S meal. The WB-PMPDs resulted in more pronounced p-AA responses compared to the SB-PMPDs. The W/S+5AA and W/S+6AA meals yielded higher p-AA responses than the W and the S meals alone.

**Table 5:** Incremental postprandial areas under the curve (iAUC 0–60 min) for the different plasma amino acids^1^.

	Products						
Amino acids	Reference	Whey	W+5AA	W+6AA	Soy	S+5AA	S+6AA
	**mmol/L**						
Alanine	2.3±0.5^c^	6.8±0.6^a^	5.6±0.5^a^	4.8±0.7^ab^	4.5±0.4^abc^	3.3±0.4^bc^	3.2±0.4^bc^
Arginine	0.8±0.1^c^	2.2±0.2^b^	2.0±0.1^b^	5.6±0.6^a^	2.5±0.2^b^	2.6±0.2^b^	5.8±0.6^a^
Asparagine	0.4±0.1^c^	2.9±0.3^a^	1.2±0.1^bc^	1.5±0.3^b^	1.9±0.3^ab^	1.1±0.1^bc^	1.1±0.2^bc^
Glutamic acid	0.3±0.1^ab^	0.6±0.1^a^	0.1±0.1^b^	0.2±0.1^ab^	0.3±0.1^ab^	0.2±0.1^b^	0.2±0.1^b^
Glutamine	0.8±0.3^c^	4.0±0.6^a^	2.5±0.4^b^	1.9±0.5^bc^	2.1±0.4^bc^	1.3±0.3^bc^	1.3±0.3^bc^
Glycine	1.4±0.7^a^	1.6±0.3^a^	0.6±0.1^a^	0.5±0.2^a^	1.8±0.3^a^	0.5±0.1^a^	0.8±0.2
Histidine	0.3±0.1^d^	2.7±0.2^a^	1.4±0.1^b^	1.3±0.1^bc^	1.4±0.1^b^	0.8±0.1^cd^	0.8±0.1^cd^
Isoleucine	0.7±0.1^e^	6.3±0.4^c^	9.4±0.5^a^	8.8±0.8^ab^	2.5±0.1^d^	7.6±0.3^abc^	7.2±0.4^bc^
Leucine	1.1±0.2^d^	9.1±0.5^b^	15.6±0.8^a^	13.7±0.8^a^	3.5±0.3^c^	13.1±0.6^b^	11.2±0.8^b^
Lysine	1.0±0.2^d^	9.0±0.5^b^	12.2±0.6^a^	12.4±0.9^a^	3.6±0.3^c^	10.0±0.5^b^	9.6±0.6^b^
Methionine	0.2±0.0^c^	1.3±0.1^a^	0.7±0.0^b^	0.7±0.0^b^	0.4±0.0^bc^	0.4±0.0^c^	0.4±0.0^c^
Phenylalanine	0.5±0.1^c^	1.3±0.1^a^	0.8±0.0^bc^	0.7±0.1^bc^	1.2±0.1^a^	0.8±0.1^b^	0.7±0.0^bc^
Proline	2.1±0.4^c^	6.7±0.4^a^	4.2±0.4^b^	3.4±0.3^bc^	4.2±0.3^b^	2.6±0.3^c^	2.7±0.4^c^
Serine	0.7±0.1^c^	3.3±0.3^a^	2.1±0.2^b^	2.1±0.2^b^	1.9±0.2^b^	1.6±0.1^b^	1.6±0.1^b^
Threonine	0.8±0.1^d^	6.0±0.5^c^	8.8±0.5^a^	8.8±0.7^ab^	2.1±0.2^d^	7.0±0.4^bc^	6.6±0.5^c^
Tyrosine	0.4±0.2^c^	1.8±0.2^a^	0.9±0.1^bc^	0.9±0.2^bc^	1.3±0.1^ab^	0.8±0.1^bc^	0.7±0.2^bc^
Valine	1.2±0.3^f^	7.8±0.4^d^	13.0±0.7^a^	13.0±0.8^ab^	3.4±0.3^e^	10.7±0.6^bc^	10.6±0.6^c^

1 All values are mean ± SEM; n = 14 (n = 13 for Soy and S+6AA; n = 12 for W+6AA). Values in the same row with different superscript letters are significantly different, P≤0.05 (ANOVA followed by Tukey’s multiple comparisons test). S (soy protein isolate); S+5AA (S, iso, leu, lys, thr and val); S+6AA (S+5AA and arg); W (whey protein isolate); W+5AA (W, iso, leu, lys, thr and val); W+6AA (W+5AA and arg).

### Plasma Incretins

**Figure 3:**
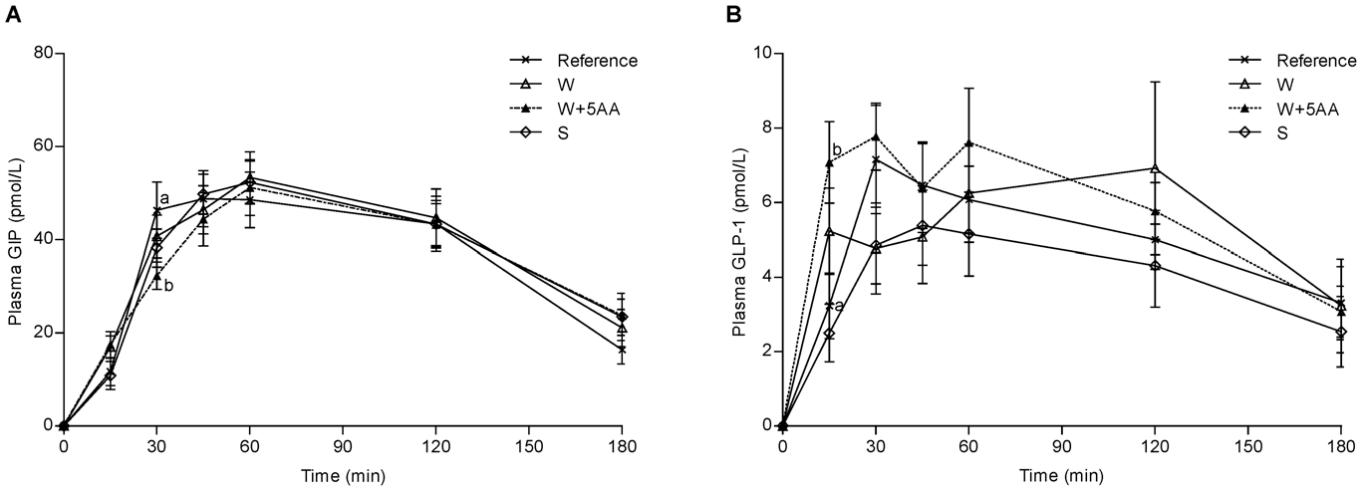
Postprandial plasma incretin responses. Mean (± SEM) incremental postprandial changes in GIP (A) and GLP-1 (B) in response to equal amounts of carbohydrates from a reference meal and the PMPD meals; n = 14 (n = 13 for S and S+6AA; n = 12 for W+6AA). Significant treatment effects (P<0.0001) but no time x treatment interactions were found for neither GLP-1 nor GIP. S (soy protein isolate); W (whey protein isolate); W+5AA (W, iso, leu, lys, thr and val).

In **Figure 3** the iAUC are shown. The GLP-1 response was higher at iAUC 0–15, 0–30 and 0–45, respectively, after W+5AA compared to the reference meal. No significant differences were found for GIP.

### Subjective Appetite Ratings and Plasma Ghrelin Responses

No significant differences were found for the AUC’s between the PMPD meals and reference meal neither for feeling of fullness, feeling of hunger, desire to eat nor for ghrelin. No treatment effects or time x treatment interactions were found (0–180 min) for either of the subjective appetite parameters. The ghrelin levels following all meals decreased to a nadir occurring at 99.5±3.0 min postprandially. Relative changes from nadir to the concentration at 180 min after commencing the PMPDs, as well as the concentration at 180 min, and the AUC 0–180 min, showed no differences following the different PMPDs and reference meals.

### Correlations

Correlations are displayed in **Table 6** **.** In the early phase, expressed as iAUC 0–15 min, the insulin responses correlated positively to the p-AA (r = 0.71, P**<**0.001), GIP (r = 0.61, P<0.001) and GLP-1 (r = 0.36, P**<**0.012) as well as to the GP (r = 0.39, P<0.001). In addition, inverse correlations were observed between early insulin (iAUC 0–15 min) and the glucose iPeak (r = −0.28, P<0.001). Moreover, the p-AA (iAUC 0–15 min) were positively correlated to the GP (r = 0.44, P**<**0.001) and inversely correlated to the glucose iPeak (r = −0.35, P**<**0.001).

**Table 6:** Spearman’s partial correlation coefficients and *P* values controlling for subjects and corresponding baseline values (two-tailed test), for the relations between plasma amino acids, plasma incretins, serum insulin (iAUC 0–15 min, respectively), and plasma glucose (expressed as GI, GP and Glucose iPeak)^1^.

		GI	GIP	GlucoseiPeak	GLP-1	GP	IGI^2^	Insulin
∑ 5 AA^3^	r	−**0.233**	**0.478**	−**0.364**	**0.376**	**0.441**	**0.621**	**0.724**
	P	**0.028**	**<0.001**	**<0.001**	**0.010**	**<0.001**	**<0.001**	**<0.001**
∑ 6 AA^4^	r	−**0.217**	**0.478**	−**0.348**	**0.376**	**0.439**	**0.645**	**0.707**
	P	**0.041**	**<0.001**	**<0.001**	**0.010**	**<0.001**	**<0.001**	**<0.001**
GIP	r	0.141	**–**	−0.038	**–**	0.123	**0.334**	**0.608**
	P	0.333	**–**	0.794	**–**	0.399	**0.019**	**<0.001**
GLP- 1	r	−0.039	**–**	−**0.286**	**–**	**0.316**	**0.335**	**0.361**
	P	0.794	**–**	**0.049**	**–**	**0.029**	**0.020**	**0.012**
II	r	−**0.588**	**0.297**	−**0.599**	0.267	**0.489**	**–**	**–**
	P	**<0.001**	**0.038**	**<0.001**	0.067	**<0.001**	**–**	**–**
Insulin	r	−0.089	**0.608**	−**0.278**	**0.361**	**0.387**	**–**	**–**
	P	0.411	**<0.001**	**<0.001**	**0.012**	**<0.001**	**–**	**–**

1 Significant correlations are shown in bold text.

2 The IGI is correlated to the amino acid (iAUC 0–45 min).

3 ∑ 5 Amino acids: Sum of iAUC of threonine, valine, isoleucine, leucine and lysine.

4 ∑ 6 Amino acids: Sum of iAUC of threonine, valine, isoleucine, leucine, lysine and arginine.

GI, glycemic index; GIP, glucose-dependent insulinotropic polypeptide; GLP-1, glucagon-like peptide 1; GP, glycemic profile; IGI, insulinogenic index; II, insulinemic index.

## Discussion

In the present study we showed that starting a meal by drinking an insulinogenic protein/AA drink significantly reduced the postprandial glycemic response to a subsequent composite meal. Interestingly, no differences were observed in insulinemic responses with respect to either iAUC 0–180 min or iPeak, although whey protein and the added AA (thr, lys, ile, leu and val) are known to be insulin secretagogues [11], [21], [22]. This effect on glycemia in the absence of differences in over-all course of insulinemia could be explained by a rapid early insulin response. Consequently, in the present study we found that the early incremental insulin response (iAUC 0–15 min) correlated negatively with the glucose iPeak, and positively with the GP. The WB-PMPD meals appeared to be somewhat more potent in lowering glycemia compared to the SB-PMPD meals. However, the SB-PMPD meals also demonstrated a positive metabolic effect and the observed glycemic reduction with soy protein is in accordance with previous studies in healthy subjects [30]. Furthermore, we found positive correlations between early p-AA (iAUC 0–15 min) and early insulin response (iAUC 0–15 min), as well as negative correlations between the p-AA and the blood glucose iPeak and the GI, respectively. Moreover, the GPs were higher following the AA-containing PMPDs compared to S, W and reference meals. Impairment of first phase insulin secretion has been recognized as an early sign of β-cell dysfunction in both T2D patients as well as in subjects with impaired glucose tolerance (IGT) [23], [24]. Interestingly, addition of essential AA to a diet in poorly regulated and elderly T2D patients improved metabolic control, lowered fasting blood glucose and insulin levels [25], [26]. Most of the insulinogenic AA trigger insulin secretion by mechanisms that differ from those that are glucose-mediated [27]. In T2D, the insulinogenic effects of AA may remain unaffected even after long-term diabetes [9], [28], indicating an interesting potential for use of proteins/AA for metabolic control of T2D. It could be hypothesized that an insulinogenic protein/AA drink could be beneficial with respect to glycemic regulation in both T2D and IGT subjects, since the insulinogenic effect of proteins and AA, appear only when co-ingested with carbohydrates. Thus, un-physiological hypoglycemic episodes that are commonly reported with drug treatment of T2D patients [29], are likely to be avoided.

Although the results in the present study are based upon acute postprandial responses, long term intake of the 5 AA on insulin resistance are known from animal studies [12] and it can be hypothesized that similar positive effects on insulin resistance and glycemic regulation might also be observed following a longer term intervention in human subjects. Additionally, the BCAA have shown to positively affect several metabolic pathways, and they were recently suggested to increase longevity in mice [30].

Interestingly, we also observed that the early GIP and GLP-1 (iAUC0–15 min) responses, correlated strongly with both the early insulin and p-AA responses. Consequently, p-AA as well as the incretins may have contributed to the insulin stimulating mechanism. Additionally, in the present study we found that the GLP-1-responses in the early postprandial phase (iAUC 0–15, 0–30 and 0–45 min), were significantly higher following W+5AA meal, compared to the reference meal. No such effects were observed with the W or the S meal, respectively, and the increased incretin secretion could possibly be ascribed the AA supplementation.

GLP-1 has been reported to display a biphasic postprandial response, with the first peak occurring already after 15–30 min [31]. Although the main part of the GLP-1 secreting L-cells are located in the distal ileum and colon, L-cells are found throughout the small intestine [32]. The L-cells in the proximal part of the gut could possibly be responsible for the early secretion, observed in the present study. Interestingly, GLP-1 does not only stimulate insulin release but has also been shown to preserve the β-cell function. Of relevance in this context is that the ability to secrete incretins are impaired in T2D patients [33], and GLP-1 analogues are increasingly used in T2D treatment [34], [35]. As judged from the present work, whey protein and certain AA may act as GLP-1 secretagogues. In addition, it has previously been shown that AA could have GIP stimulatory effects [36], [37]. Furthermore, i*n vitro* studies of isolated pancreatic islets from mouse have indicated that whey protein exerts its insulinogenic effect by elevation of p-AA and GIP and GLP-1 [38]. In the present study we found no significant differences on GIP secretion following the W, the W+5AA, nor the S meal, in comparison to the reference meal. We have previously observed that whey protein, but not an AA-mix per se, enhanced GIP secretion when co-ingested with 25 g of carbohydrates [11]. However, in the present study the composite meal contributed with the double amount of available carbohydrates (50 g), 10 g of additional fat and the serving sizes of the whey protein was half of that served in the previous study (9 vs.18 g). Both carbohydrates and fat are known to stimulate GIP secretion [39], and in the present study the protein/AA-induced effect on GIP could be masked by the effect from the fat and carbohydrates in the composite meal.

In the present study, we also aimed to investigate the possible enhancing effect on insulin secretion by adding arg to the 5AA-mix (thr, lys, ile, leu and val). Arg has been reported to enhance the pancreatic insulin secretion when co-ingested with leu [14]. However, in the present study, no further increase in insulinemic response was observed with arg added compared to that seen with the 5AA-mix.

Low grade inflammation is suggested as one contributing factor in the development of T2D [40], [41]. Markers of low-grade inflammation appears to be promoted by high postprandial blood glucose levels [42], implying that tight glycemic regulation may be advantageous in this context. Interestingly, it has been shown that high GI meals (white wheat bread or glucose) are more potent drivers of acute inflammation (nuclear factor κB) compared to low GI meals (pasta) in young healthy subjects [43]. Addition of the PMPD to a composite meal in the present study, significantly lowered the glycemic excursion and could therefore possibly lower postprandial inflammation, but further research in this field is required.

No significant differences were found for the subjective appetite parameters or ghrelin in the present study. Both whey and soy proteins have previously been ascribed satiating properties [15], [44]. It has also been postulated that whey protein induce higher short term satiety than carbohydrates and fat, respectively [45]. The lack of satiating effects of the test meals in this study is probably due a small number of participants, and to the low overall protein content.

There were some limitations of the study that need to be addressed. The female participant’s menstrual cycle stages were not screen for and possible influences of hormonal changes cannot be ruled out. The amount of protein in W+6AA and S+6AA was 0.7 g higher compared to the other meals, due to the arginine addition. Increased protein content could be expected to increase the insulinogenic effect. However, no such effect was found. Another limitation is that the entire volume of water (250 ml) was taken along with the sandwiches in the case of the reference meal, not as a pre-meal drink. However, previous studies have shown that neither volume nor timing of drinking the water affects the postprandial glycemia [46].

### Conclusions

The reduction in postprandial glycemia seen after intake of pre-meal drinks consisting of whey and soy protein, with or without supplementation of an insulinogenic AA-mix (iso, leu, lys, thr and val) was substantial (up to about 47%) and was mediated by a significant increase in the very early postprandial insulin response. The early insulin response correlated to the early appearance of specific AA and GLP-1 in plasma, indicating the involvement also of this incretin**.** It is concluded that the enclosure of specific proteins and AA in a pre-meal drink offers an interesting strategy to attenuate postprandial glycemic excursions and promote release of the antidiabetic incretin GLP-1.

## Supporting Information

Protocol S1 Trial protocol
(DOCX)

Flow Diagram S1 Study Design (CONSORT Diagram)
(DOC)

Checklist S1 CONSORT checklist
(DOC)
