# Supplementary material for: Effects of Pre-Meal Drinks with Protein and Amino Acids on Glycemic and Metabolic Responses at a Subsequent Composite Meal
Source: PLoS One. 2012 Sep 19;7(9):e44731. doi: 10.1371/journal.pone.0044731 (PMC3446992; doi:10.1371/journal.pone.0044731)
Supplement: Protocol S1 — Trial protocol (DOCX) [file pone.0044731.s001.docx]

**An English translation of the Swedish trial protocol**

**“Postprandial effects on blood glucose, hormone and appetite responses following different foodstuffs”**

**Background**

It is well documented that glucose and insulin responses differs following various carbohydrate-rich foods intake [[1](#_ENREF_1)]. This discrepancy can be explained by different food factors that affect the digestion and absorption rate of the carbohydrates in the gut and small intestine. So-called “rapid” foods results in high levels of insulin after meals, which are increasingly discussed as a risk factor for the development of several of our lifestyle diseases such as diabetes, cardiovascular disease and obesity. Several epidemiological studies show a correlation between diets characterized by “slow” foods and the protection against development of type 2 diabetes [[2](#_ENREF_2),[3](#_ENREF_3)]. There is an increasing interest to improve the nutritional qualities of carbohydrates in foods and diets. A diet rich in food containing “slow” carbohydrates is especially beneficial for diabetics, who then need less insulin to match the blood glucose levels, and for subjects with hyperlipidemia where meals with “slow” foods have shown to reduce plasma lipids. Recent data indicates that oxidative stress might contribute to insulin resistance, and foods with high content of antioxidants could by hypothesize to have a preventive potential. Additionally, food proteins have been shown to affect the insulin response differently after a meal [[4](#_ENREF_4)]. A dietary intervention that reduces the levels of circulating insulin, thereby improving insulin sensitivity, could be postulated to reduce the development of the insulin resistance syndrome and would be of great value to public health.

**Objective**

The aim of the present studies is to evaluate the effect of carbohydrates, proteins and bioactive compounds in approved foods and food additives on blood glucose and hormone responses and appetite after a meal. The studies are based on acute meals trial in healthy volunteers. Postprandial levels of blood glucose, hormones such as insulin and GLP-1, GIP, inflammation markers and subjective satiety and biomarkers that can be linked to appetite regulation (eg ghrelin) is recorded in capillary and venous blood, if necessary. Subjective satiety and hunger data for each study occasion.

**Description of products and meals**

The test products are approved foods and food additives. The total number of foods included in each test series will be maximum 6 meals, and additionally two glucose beverages will be served as reference meals. The test products will be served as breakfast meals in randomized order. Meals are standardized to 25-50 g available starch [[5](#_ENREF_5)]. In all cases, these are common foods and food additives, i.e. *food grade.*

**Type of study**

The studies are acute. Subjects are instructed to eat a standardized meal, in the evening (between 21.00 and 22.00) prior to every test day, consisting of white wheat bread slices and an optional drink. Thereafter they are instructed to avoid eating and drinking anything but small amounts of water until the start of the test. Additionally they are asked to avoid alcohol, excessive physical activity and food rich in dietary fibers the day before each test. Test meals are served as breakfast in random order. Before the meal commences in the morning, a blood sample (time 0) is taken to determine fasting blood glucose and insulin levels. The meals are consumed in approximately 12 minutes. Capillary and venous blood samples are then taken at 15, 30, 45, 60, 90, 120 and 180 min after the meal started. The subjects are also asked to repeatedly fill in their subjective feeling of fullness, hunger and desire to eat*.* Each person takes a maximum of six test meals and two references. Meals are taken with at least one week apart.

**Test subjects**

Test subjects (about 15 subjects) mainly consist of graduate students and students at Lund University and they are not in dependence of the investigator. Many have participated in previous, similar, studies. Advertising is done on local bulletin boards and contacts are made through e-mail or phone. In addition, test subjects spread information about our research and the need of people helping to interested contacts. Research subjects must be healthy and normal weight, aged 18 years or upwards. They may at any time terminate the study without giving any reason.

**Analyses and blood volumes**

Blood samples are analyzed for blood glucose and hormones such as insulin, GIP, GLP-1, ghrelin and inflammation markers. In addition, subjective satiety and hunger responses are analyzed using pen and paper.

The amount of blood collected at each time point based on the following calculation:

Capillary blood for glucose: 50µl

Serum (capillary) insulin: 1 tube 0.5 ml of blood

Serum (venous) for hormones and inflammatory markers: a pipe á 3,5 ml of blood

Plasma (venous) for hormones and inflammatory markers: a pipe á 4 ml of blood

This gives about 65 ml of blood per test day and corresponds to 250 ml per person for the entire test series.

**Risks and potential incidents**

Possible risks of collecting blood samples are considered very low. The risk for incidents associated with the input experimental product does not exist, where the subjects are asked before inclusion on the prevalence of food allergies and / or intolerances.

**Registration of the experimental data**

All data will be recorded on paper and in an anonymised form for use in computer programs for statistical analysis.

**References**

1. Bjorck I, Liljeberg H, Ostman E (2000) Low glycaemic-index foods. British Journal of Nutrition 83 Suppl 1: S149-155.

2. Salmeron J, Ascherio A, Rimm EB, Colditz GA, Spiegelman D, et al. (1997) Dietary fiber, glycemic load, and risk of NIDDM in men. Diabetes Care 20: 545-550.

3. Salmeron J, Manson JE, Stampfer MJ, Colditz GA, Wing AL, et al. (1997) Dietary fiber, glycemic load, and risk of non-insulin-dependent diabetes mellitus in women. JAMA 277: 472-477.

4. Nilsson M, Stenberg M, Frid AH, Holst JJ, Bjorck IM (2004) Glycemia and insulinemia in healthy subjects after lactose-equivalent meals of milk and other food proteins: the role of plasma amino acids and incretins. American Journal of Clinical Nutrition 80: 1246-1253.

5. Holm J, Bjorck I, Drews A, Asp NG (1986) A Rapid Method for the Analysis of Starch. Starch-Starke 38: 224-226.
