# Supplementary material for: Effects of Pre-Meal Drinks with Protein and Amino Acids on Glycemic and Metabolic Responses at a Subsequent Composite Meal
Source: PLoS One. 2012 Sep 19;7(9):e44731. doi: 10.1371/journal.pone.0044731 (PMC3446992; doi:10.1371/journal.pone.0044731)
Supplement: Flow Diagram S1 — Study Design (CONSORT Diagram) (DOC) [file pone.0044731.s002.doc]

**CONSORT 2010 Flow Diagram**

**Allocation**

**Analysis**

**Follow-Up**

**Enrollment**

Assessed for eligibility (n=14)

Excluded (n=0)

♦  Not meeting inclusion criteria (n=0)

♦  Declined to participate (n=0)

♦  Other reasons (n=0)

Analysed (n=14)
♦ Excluded from analysis (give reasons) (n=0)

Lost to follow-up (give reasons) (n=0)

Discontinued intervention (give reasons) (n=0)

Allocated to intervention (n=14)

♦ Received allocated intervention (n=14)

♦ Did not receive allocated intervention (give reasons) (n=0)

Randomized (n=14)
